# MDED-Framework: A Distributed Microservice Deep-Learning Framework for Object Detection in Edge Computing

**DOI:** 10.3390/s23104712

**Published:** 2023-05-12

**Authors:** Jihyun Seo, Sumin Jang, Jaegeun Cha, Hyunhwa Choi, Daewon Kim, Sunwook Kim

**Affiliations:** Artificial Intelligence Research Laboratory, ETRI, Daejeon 34129, Republic of Korea; jsm@etri.re.kr (S.J.); jgcha@etri.re.kr (J.C.); hyunwha@etri.re.kr (H.C.); won22@etri.re.kr (D.K.); swkim99@etri.re.kr (S.K.)

**Keywords:** multi-object detection, edge computing, deep learning, distributed system, software framework

## Abstract

The demand for deep learning frameworks capable of running in edge computing environments is rapidly increasing due to the exponential growth of data volume and the need for real-time processing. However, edge computing environments often have limited resources, necessitating the distribution of deep learning models. Distributing deep learning models can be challenging as it requires specifying the resource type for each process and ensuring that the models are lightweight without performance degradation. To address this issue, we propose the Microservice Deep-learning Edge Detection (MDED) framework, designed for easy deployment and distributed processing in edge computing environments. The MDED framework leverages Docker-based containers and Kubernetes orchestration to obtain a pedestrian-detection deep learning model with a speed of up to 19 FPS, satisfying the semi-real-time condition. The framework employs an ensemble of high-level feature-specific networks (HFN) and low-level feature-specific networks (LFN) trained on the MOT17Det dataset, achieving an accuracy improvement of up to *AP*_50_ and *AP*_0.18_ on MOT20Det data.

## 1. Introduction

Multiple object detection is a computer vision field that involves analyzing images and videos to extract information about object classes and their locations. It has been extensively studied in various domains, including autonomous driving [1,2], anomaly detection [3,4], surveillance [5,6], aerial imagery [7,8], and smart farming [9,10]. By utilizing artificial intelligence algorithms, research in this this field aims to address challenging detection problems. However, with the rapid increase in the amount of real-time video data acquired from sensors and IoT devices, there is a growing need for distributed computing to process those data effectively. Consequently, there is an increasing demand for the development of deep learning models optimized for distributed computing environments while maintaining detection accuracy.

Distributed processing techniques via cloud computing have been used to address high computational demands and resource constraints in deep learning models. However, cloud computing suffers from limited bandwidth for data transfer and significant latency when transferring large amounts of video data [11,12]. To address these issues, edge computing is emerging as a solution, where deep learning servers are placed closer to the physical locations where video data is generated, allowing data to be processed and analyzed at the edge [13,14]. Edge computing is a horizontal architecture that distributes computing resources on edge servers closer to users and edge devices.

However, there are several issues that need to be addressed in order to proceed with the distributed processing of deep learning in an edge computing environment that solves the shortcomings of cloud computing. First, we need a framework that can automatically configure and maintain the environment of various devices. If a new device added to the cluster has enough GPU and memory, it can deploy a large deep learning model and make inferences. However, for a smartphone or micro device, deploying a large deep learning model may cause inferences to experience delays or failures. In traditional frameworks, cluster managers monitor this and manually deploy models accordingly. However, as the number of devices connected to the cluster increases, it becomes very difficult to manage the deployment of models manually. Therefore, if automated microservice deployment is possible in an edge computing cluster environment, it would make life easier for service managers. Additionally, microservices need to be able to scale in and out as user requests increase or decrease in volume. Traditionally, administrators manually deploy and release microservices to devices using spare resources, but this is highly inefficient and users may experience service inconvenience due to the difficulty of instant microservice processing. Automated microservice deployment can monitor the resources of edge devices and dynamically scale them in and out, providing convenience to service users.

Second, there is a need for a framework that is optimized for distributed edge environments that includes improving the accuracy of deep learning models. The aim of deep learning models is to achieve high performance, which typically requires the use of large models. However, such models may not be suitable for low-resource edge environments. To overcome this challenge, lightweight models can be used instead. Yet, training such models using traditional methods [15] may lead to overfitting, long training times, and even decreased performance. Therefore, a distributed computing environment can be utilized, with multiple edge devices connected, to achieve good results even from lightweight deep learning models with low performance.

In this paper, to address the above problems, we propose a microservice deep-learning edge detection framework (MDED-Framework) that applies an architecture suitable for distributed processing and can improve performance by parallelizing existing learned object detection models.

The contributions of the proposed framework are as follows:

- First, it supports efficient multi-video stream processing by analyzing the resources in an edge cluster environment. It supports flexible scale in and scale out by periodically detecting resource changes in the cluster. It also minimizes delay through efficient distribution of tasks.
- Second, we constructed high-level feature network (HFN) and low-level feature network (LFN) networks that lighten the scaled YOLOv4 [16] model. The model lightweighting based on the training features of the deep learning model provides improved detection accuracy even on more complex data than the trained set.
- Third, we implemented the deep learning model ensemble in a distributed environment to maximize the benefits of distributed processing. In addition to improving processing speed, object detection can continue even when some services fail.
- Furthermore, we provide a web-based video analysis service to provide users with an easy and convenient object detection service. Through the Rest API, users can easily access functions such as selecting video resolution, which is one of the factors affecting detection accuracy, uploading videos for detection, and checking deep learning results.

The remainder of the paper is organized as follows. Section 2 explains the framework and deep learning models associated with this study. Section 3 offers an explicit explanation of the proposed framework and its methodology. Section 4 illustrates the results of the experiment. Finally, Section 5 concludes the paper and identifies avenues for future research.

## 2. Background

The first part of this section describes deep learning frameworks that operate in edge computing environments. The second part describes the types and functions of neck structures used to enhance features in CNN-based vision deep learning, and the last part describes deep learning models that consider resolution parameters to enhance accuracy.

### 2.1. Deep Learning Framework on Edge Computing

The utilization of deep learning has changed as its structure has transformed from cloud computing to edge computing. As deep-learning-based object detection is linked to various advances in the field, edge device-based platforms have emerged.

Sassu A. et al. [17] proposed a deep-learning-based edge framework that can analyze multi-streams in real time. Docker-based services are structured to be processed independently, and two example applications are shown. While [17] focuses on improving the performance of CPUs and GPUs, the end goal of deep learning applications is to improve the accuracy of the model. In this paper, we present a model that is efficient in a distributed environment and also performs well on data not used for training, focusing on both processing speed and model accuracy. Kul S. et al. [18] proposed a new means of tracking specific vehicles on a video stream collected from surveillance cameras. Data on microservices between networks are sent and each service extracts the vehicle type, color, and speed, and combines these features. Apache Kafka [19] was used to introduce a system that can offer feedback on real-time queries. Houmani Z. et al. [20] proposed a microservice resource-management scheduling method for deep learning applications that overview edge cloud. The study proposes a deep learning workflow architecture that divides cloud resources into three categories (non-intensive, low-intensive, and high-intensive) based on CPU, memory, storage, or bandwidth requirements, and which uses distributed pipelining. The study reported improved processing speeds by 54.4% for edge cloud compared to cloud-based scenarios running at 25 frames per second. In this paper, we present a resolution selector that allocates edge cloud resources according to the resolution of the image, to consider the detection accuracy of the deep learning model and how to efficiently distribute processing without compromising the resolution, which is one of the important factors of the deep learning model. Li J. et al. [21] proposed a hierarchical architecture that improves deep learning performance for vision-related processes by using multitasking training and balancing the workload. The study improved mAP for pedestrian detection and person re-identification tasks. It also introduced a simulation of indoor fall detection. Xu Z. et al. [22] proposed a real-time object detection framework using the cloud-edge-based FL-YOLO network. The FL-YOLO network adds a depth-wise separable convolution down-sampling inverted residual block to Tiny-YOLOv3 and consists of a framework that can train and validate coal mines; it uses reduced model parameters and computations. Chen C. et al. [23] introduced a new architecture that processes re-identification problems that occur when personal information issues arise as data is sent via the cloud. The architecture is processed on the AIoT EC gateway. The study designed an AIoT EC gateway that satisfies the relevant resource requirements using a microservice structure with improved process speeds and latency as the number of services increases.

### 2.2. CNN-Based Vision Deep Learning Neck Structure

Traditional image processing techniques sort the features of an image into two main categories: high-level features and low-level features. High-level features, also known as global features, refer to the overall information of an image (texture, color, etc.). These features are usually found in layers close to the input image in the structure of the deep learning model. Low-level features, also known as local features, are localized information in the image (edges, corners, etc.). Unlike high-level features, low-level features reside in the layers of the deep learning model structure that are furthest from the input image.

According to [24], humans typically recognize objects through high-level features in an image, while deep learning models detect objects through low-level features acquired through a series of operations. This means that deep learning models cannot achieve high detection accuracy by using only high-level features. However, using a large number of low-level features to increase accuracy leads to another problem: overfitting. This means that only the low-level features extracted from the data used for training have high accuracy. In deep learning architecture, the neck structure was created to solve these problems by fusing high-level features with low-level features to improve accuracy.

The neck structure is located between the backbone structure, which extracts features from the detection network, and the head structure, which determines the existence of an object through regression. The neck structure is divided into two types depending on whether or not a pyramid structure is used to fuse low-level and high-level features. A pyramid structure refers to a structure that is computed by fusing feature maps of different sizes obtained by passing through convolution layers. Some of the most well-known neck structures that create pyramid structures are the Feature Pyramid Network (FPN) [25], PAN [26], NAS-FPN [27], BiFPN [28], etc. FPN utilizes an upsampling process where the feature map obtained from the backbone in the order of high-level features to low-level features is recomputed in the order of low-level features to high-level features. This process allows the deep learning model to perform well by referring to a wider range of features. PAN is a structure that adds one more bottom-up path to the FPN structure, enriching the low-level features that can directly affect the accuracy of deep learning. NAS-FPN utilizes the NAS structure to build an efficient pyramid structure on a dataset. Bi-FPN performs bottom-up and top-down fusion through lateral connections.

On the other hand, there are also neck structures that fuse high-level features with low-level features without using a pyramid structure. Structures such as SPP [29], ASPP [30], and SAM [31] utilize specific operations on the feature map to obtain features of different sizes. SPP can obtain global features by applying max-pooling to feature maps of various sizes; its biggest advantage is that it does not require the input image size to be fixed or transformed for deep learning structures, because it operates directly on the feature map. Unlike SPP, which is a type of pooling, ASPP is a type of convolution and is characterized by expanding the area of the convolutional operation, which usually exists in a 3 × 3 size, so that a wider range can be considered. In this paper, the authors apply the scaling factor rate so that various receptive fields can be viewed. In addition, an expanded output feature map can be generated without increasing the amount of computation, and detailed information about the object can be acquired efficiently. SAM is a kind of optimizer; when calculating the loss, it optimizes the loss value while converging to the flat minima to maximize the generalization performance.

The traditional neck structure fuses low-level and high-level features in one network to improve accuracy in an end-to-end manner. This method can create a high-accuracy model in a single-server environment, but it is not suitable for environments with many variables, such as cloud edge environments, because the size of the model is a limitation. Therefore, in this paper, we add the neck structure into HFN, a network specializing in high-level features, and LFN, a network specializing in low-level features, and use ensemble techniques to compensate for the reduction in accuracy.

### 2.3. Image Resolution-Related Deep Learning Models

There are variations of deep learning models that utilize the neck structure described above, whereas some networks consider the resolution of the input image. The EfficientDet [28] network is a model constructed using the EfficientNet [32] model as a backbone. EfficientNet explains that there are three existing ways to increase the accuracy of the model: increasing the depth of the model, increasing the number of filters in the model, and increasing the resolution of the input image. The authors aimed to achieve better performance by systematically analyzing the above three variables, and designed a more efficient model using the compound method. In the EfficientDet network, they proposed a BiFPN structure, which is a variation of the existing FPN structure, and constructed feature maps with different scales through a bidirectional network, which is a resolution-dependent structure, to learn richer features.

The PP-YOLO [33] network does not present a new detection method, but it combines several techniques that can improve the accuracy of the YOLOv3 [34] network, resulting in lower latency and higher accuracy than existing models. PP-YOLO achieved acceptable performance for images with resolution sizes of 320, 416, 512, and 608, and utilizes a similar structure to the existing FPN.

Scaled YOLOv4 [16] is a model scaling network based on the existing YOLOv4 network. Based on YOLOv4-CSP, the authors developed the Scaled-YOLOv4—large and Scaled-YOLOv4—tiny models, with a modified structure, by combining the three parameters (depth, width, and resolution) proposed by EfficientNet. These models are characterized by better control of computational costs and memory bandwidth than existing models. Depending on the resolution, the scaled YOLOv4 model showed improved performance compared with the EfficientDet model, as well as fast inference speed. In this paper, we modified the network based on the Scaled YOLOv4 model with good performance to deploy the model smoothly in the edge computing environment, and constructed a framework that can target and process images with resolutions of 640, 896, and 1280.

## 3. MDED Framework

### 3.1. System Architecture

**Figure 1:**
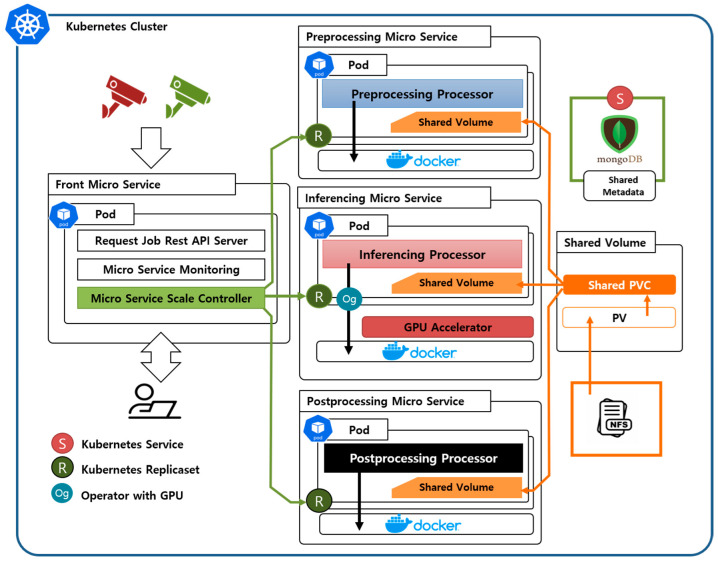
Illustration of the overall configuration of the MDED framework.

As shown in Figure 1, the MDED framework consists of microservices that perform video object detection, a MongoDB service, and persistent volumes. To make the framework suitable for distributed processing, a cluster consists of a set of multiple individual nodes. The nodes’ environments vary widely, and microservices are automatically deployed that are appropriate for each node’s resources. Microservices are built on top of Docker [35] containers and provide services in the form of Kubernetes [36] pods, the smallest unit that can be deployed on a single node. Microservices are organized into four types: front microservices, preprocessing microservices, inferencing microservices, and postprocessing microservices. The front microservice monitors the resources (CPU, GPU) inside the cluster and coordinates the deployment of other microservices. The front microservice also provides users with a web-based video object detection API. The preprocessing microservice splits the user-input video file into frames and performs preprocessing tasks on CPU resources to transform the video to the user-specified resolution. The inferencing microservice distributes video frames to the high-level feature-specific network (HFN) and low-level feature-specific network (LFN) to obtain detection results from each network. GPU resources are prioritized, and if they are insufficient, object detection is performed using CPU resources. The postprocessing microservice ensembles the results obtained through distributed processing in the inferencing microservice. It calibrates the results to achieve better detection accuracy than can be achieved with a single deep learning model, and performs visualization tasks such as displaying the detection results on the screen. Each microservice is described in detail in later sections: Section 3.1.1, Section 3.1.2 and Section 3.1.3.

The microservices operate independently and share the metadata generated by each microservice through MongoDB, which also prevents more than one microservice from accessing the same data at the same time. Each microservice references input data (video) and output data (class, bounding box) via NFS, which is a shared volume. NFS is created through the binding of a PV to a PVC, and as user needs change, a new PVC can be created to provide flexible connectivity to other PVs with the appropriate storage capacity.

#### 3.1.1. Front Microservice

The front microservice is responsible for communicating directly with users and showing work progress and results. In addition, it can monitor the resources and workload of edge nodes connected to the cluster to create an environment that handles data flexibly. The front microservice periodically updates the information about the overall resource and usable resources of the edge node, and automatically determines whether to generate new microservices as it receives new input data. Moreover, the flexible distributed processing environment can be constructed by monitoring the processing workload and adjusting the scalability of microservices.

The front microservice performs the following functions:

- Rest API server: receives video data and user metadata that are used to build inference pipelines. The inference results stored in NFS are displayed on the HTTP API to provide user-friendly services. The Rest API server is implemented through the micro-framework Flask [37].
- Microservices resource monitor (MRM): monitors the available resources and current workload on the edge nodes. The obtained information is passed to the Microservices scale controller to configure the optimal microservices operating environment based on the resource state and to configure an efficient distributed processing environment.
- Microservices scale controller (MSC): the results of MRM are used to adjust the number of microservices to distribute processing jobs. If the workload is increasing, the MSC uses information obtained through MRM to determine whether microservices can increase or not. As the workload decreases, the resource release process begins to gradually reduce idle microservices. Algorithm 1 introduces the resource allocation/release algorithm for MSC.

**Algorithm 1.** Resource allocation/release algorithm.**Input**: Microservice monitoring information (number of states for each task) **Output**: the newly created process pod or returned resourcesDef Microservices Scale Controller While(True): Sleep(5) Processes_lists = Microservice Monitoring() Preprocess_ratio = The number of Enqueue/The number of Preprocess Inference_ratio = The number of preprocess_complete/The number of inferences Postprocess_ratio = The number of inference_complete/The number of postprocess //CPU loop While preprocess_ratio, postprocess_ratio close to threshold: If preprocess_ratio, postprocess_ratio > threshold: //Scale-out If there are sufficient CPU resources to add new pods The number of replicaset += 1 elif preprocess_ratio, postprocess_ratio < threshold: //Scale-down The number of replicaset −= 1 Microservices Scale Controller(preprocess) or   Microservices Scale Controller(postprocess) //GPU loop Resolution_lists = Microservices Monitoring() Gpu_var = cuda.device_count() While inference_ratio closes to threshold If inference_ratio > threshold:          //Scale-out If gpu_var > The number of inference The number of GPU inference replicaset += 2  Elif inference_ratio < threshold:          //Scale-down  The number of GPU inference replicaset −= 2  Microservice Scale Controller(inferencing)

#### 3.1.2. Preprocessing Microservice

**Figure 2:**
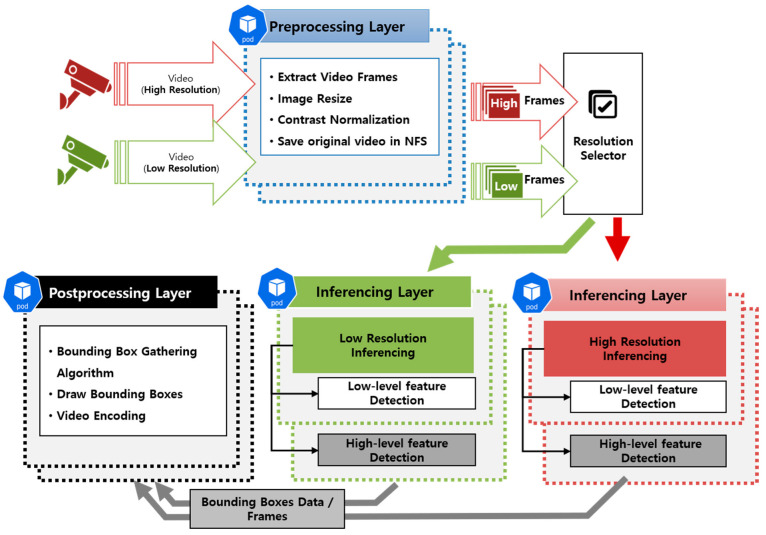
Illustration of the overall flow of the proposed method.

Figure 2 illustrates the processing flow of the MDED framework, which consists of several microservices designed to perform specific tasks. The framework includes a preprocessing layer, an inference layer, and a postprocessing layer, each implemented as a separate microservice. The first microservice, the preprocessing microservice, handles the video data input from the user via the front microservice on the web. It obtains video frame information from the processed video data. The preprocessing microservice splits the video data source file into images of 30 frames per second. In the process, the image is processed according to whether the user wants to use a specific resolution of the image for detection, or wants to use only a part of the image for detection. The preprocessing microservice obtains information about the processed images and classifies them according to resolution for efficient resource utilization. Resolution is one of the variables listed in [28] that can improve accuracy, and the resolution selector is responsible for matching images with the best resource environment to run without degrading their resolution. The resolution selector runs in two categories: low resolution (640p, 896p) and high resolution (1280p) to help prioritize the distribution of low-resolution video to less resourceful nodes and high-resolution video to more resourceful nodes.

#### 3.1.3. Inferencing Microservice

**Figure 3:**
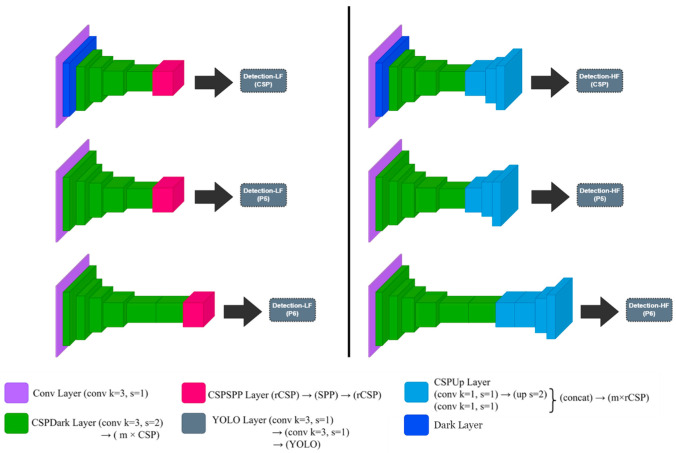
LFN and HFN of csp, p5, and p6 architecture.

The inferencing microservice supports multi-object inference using deep learning models. The inferencing microservice consists of two networks, a high-level feature-specific network (HFN) and a low-level feature-specific network (LFN), which modify the Scaled YOLOv4 [16] network according to the features in the image. Additionally, since the Scaled YOLOv4 network on which it is based considers the resolution of the input image, we modified the system to fit the Scaled YOLOv4-csp, Scaled YOLOv4-p5, and Scaled YOLOv4-p6 networks. Figure 3 shows the structure of the LFN and HFN according to csp, p5, and p6.

HFN and LFN are networks with improved performance in edge environments for pedestrian objects [38]. Scaled YOLOv4 and high-performance networks attempt to improve accuracy by using a neck structure. However, the number of parameters increases, and the computational demand increases when fusing low-level and high-level occurs multiple times. This causes the model to grow larger and larger in size, making it difficult to deploy deep learning models in edge environments where resources may be insufficient. It is also difficult to scale out and scale down the model for flexible distributed processing. Therefore, we wanted to modify the Scaled YOLOv4 model to be suitable for use in distributed processing environments.

HFN and LFN are networks that specialize in high-level features and low-level features, which in Scaled YOLOv4 serve as inputs to the top-down pathway and bottom-up pathway performed by the PANet [26]. In the case of an HFN, the network is trained by acquiring and optimizing the features acquired around the input of the backbone. However, high-level features cannot be expected to be highly accurate for training deep learning models, so we applied FPN to further fuse them with low-level features. The LFN is a network that strengthens the last layer of the backbone network, which is the part where low-level features mostly gather. We added an SPP [29] layer after convolution to strengthen the global feature information of the low-level features, which also serves to prevent overfitting.

As shown in Figure 2, the high-level feature-specific network and the low-level feature-specific network fall under the same process, referred to as the inference layer, and are distributed to different pods. The inputs are image frames whose resolutions are classified by a resolution selector, and the inference microservices prioritize images with resolutions appropriate to the environment in which they are performing. HFN and LFN detect objects in parallel and store the results in shared storage.

#### 3.1.4. Postprocessing Microservice

The postprocessing microservice is responsible for assembling the results obtained from the inferencing microservices and utilizing CPU resources to extract useful information. Additionally, if the user wishes to view the results, the postprocessing microservice offers the ability to display the bounding boxes directly on the image. The final detection results represent the objects obtained, assembled or encoded into a video.

As shown in Algorithm 2, the bounding box gathering algorithm is used to obtain the final meaningful bounding boxes from the HFN and LFN bounding boxes. This algorithm calculates the intersection over union (IoU) of the bounding boxes *R_High_* from the HFN and the bounding boxes *R_low_* from the LFN. If the IoU ratio is close to 1, the boxes are likely to represent the same object. The formula for calculating the IoU is presented below.

(1) $$Intersection\;over\;Union\left(IoU\right)=\frac{Intersection\;}{Area_{A}+Area_{B}-Intersection}$$

**Algorithm 2.** Bounding box gathering algorithm.**Input**: HFN detection results $R_{High}=\left\{\;b_{h1},\;b_{h2}\cdots ,\;b_{hn}\;\right\}$, LFN detection results $R_{low}=\left\{\;b_{l1},\;b_{l2}\cdots ,\;b_{ln}\;\right\}$ **Output**: Ensembled detection results $R_{Ensembled}=\left\{\;b_{e1},\;b_{e2}\cdots ,\;b_{en}\;\right\}$$R_{Ensembled}$ ← [] For $b_{h}$ in $R_{High}$:                    $R_{Ensembled}\;\leftarrow \;b_{h}$  $Area_{h}=\left(Top_{y_{b_{h}}}-Bottom_{y_{b_{h}}}\right)\times \left(Bottom_{x_{b_{h}}}-Top_{x_{b_{h}}}\right)$  For $b_{l},$ in $R_{low}$:    $Area_{l}=\left(Top_{y_{b_{l}}}-Bottom_{y_{b_{l}}}\right)\times \left(Bottom_{x_{b_{l}}}-Top_{x_{b_{l}}}\right)$    $Top_{Inter_{x}}=\max\left(Top_{x_{b_{h}}},\;Top_{x_{b_{l}}}\right)$    $Top_{Inter_{y}}=\max\left(Top_{y_{b_{h}}},\;Top_{y_{b_{l}}}\right)$    $Bottom_{Inter_{x}}=\max\left(Bottom_{x_{b_{h}}},\;Bottom_{x_{b_{l}}}\right)$               $Bottom_{Inter_{y}}=\max\left(Bottom_{x_{b_{h}}},\;Bottom_{x_{b_{l}}}\right)$    $Area_{inter}=\max\left(0,\;Bottom_{inter_{x}}-Top_{inter_{x}}+1\right)\;\;\;\;\;\;\;\times \max\left(0,\;Bottom_{inter_{y}}-Top_{inter_{y}}+1\right)$    $Area_{Union}$ = $Area_{h}+Area_{l}$ If IoU > threshold and IoU ≤ 1: Already detected pedestrian, stop. If $b_{l}$ is newly detected pedestrian:                 $R_{Ensembled}\;\leftarrow \;b_{l}$

## 4. Results

This section describes the dataset and accuracy metrics used to measure the accuracy of the inference microservice. It provides details of the experiments, and reports the results of the distributed processing time measurements.

### 4.1. Datasets and Details

**Figure 4:**
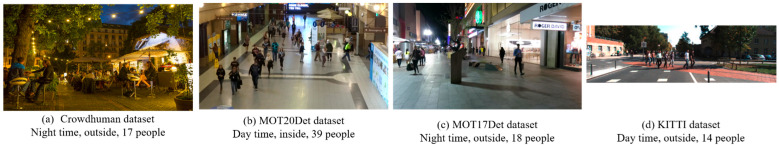
A dataset commonly used for pedestrian detection.

The datasets used to measure the accuracy of pedestrian object detection in this experiment were MOT17Det [39] and MOT20Det [40]. Various datasets contain pedestrian objects, such as KITTI [41] and CrowdHuman [42]. However, the MOTDet dataset was the only dataset with prior research on the relationship between datasets (MOT17Det and MOT20Det), so we used it as the training and test data to measure the general accuracy of the network. The MOT17Det validation and MOT20Det validation sets available on the MOTChallenge website were not used, due to authentication issues. Figure 4 shows examples of images from various datasets that are commonly used in pedestrian detection. Dataset 4-(b) is known to cover more complex and diverse situations than dataset 4-(c), and was used as test data in this experiment to assess the general performance improvement of deep learning. Table 1 shows the specific information of dataset 4-(c) (MOT17Det train) used for training and dataset 4-(b) (MOT20Det train) used for testing.

For the HFN and LFN, we extracted only pedestrian data from the MOT17Det training data and used these for training, keeping the ratio of training and validation datasets at 7:3. To measure the accuracy after applying the ensemble technique, the test dataset was the MOT20Det train dataset. Only the bounding boxes corresponding to pedestrians were extracted and used as ground truth. The images in the training and test datasets were changed to the resolutions supported by the underlying network, Scaled YOLOv4: 640 (CSP), 896 (p5), and 1280 (p6).

We trained a high-level feature-specific network and a low-level feature-specific network with a resolution of 200 epochs, a batch size of 2, and a learning rate of 0.001. Each model was implemented using Pytorch and trained on an NVIDIA GTX 3090.

### 4.2. Experimental Results

Our experiments focused on the execution time and accuracy of the proposed framework. For the preprocessing microservice and postprocessing microservice, we found that processing did not take more than 30 m/s per image, which satisfies the real-time requirement. As for execution time, this paper focused on the execution time of the deep learning model because it is most dependent on the inference of the deep learning model utilizing the GPU.

In addition, since the transfer speed of files and images may vary depending on the configuration of the microservice environment, we excluded the transfer time consumed by file transfer when measuring the results.

Table 2 shows the results for the number of parameters used by the deep learning models in the proposed framework, the number of layers pruned, and the processing speed. Since HFN and LFN are processed in parallel, we adopted the value of the lower FPS of the two networks. The results show that the inference network in the proposed framework can process on average up to two frames per second faster. We were also able to remove a certain number of parameters in the model, removing two to three million parameters.

The FPS of the Scaled YOLOv4 (p6) model was the same as that of the original model, but there was a significant difference in accuracy. We used average precision (AP) as a metric to measure the accuracy of object detection, and precision and recall metrics to check how well the model learned. In the field of object detection, precision and recall are calculated through the IoU value of similarity between the ground truth bounding box and the predicted bounding box. The precision and recall metrics are shown below. The area under the precision and recall curves, measured by dividing them by a certain interval, is called AP, and is used to represent the accuracy of a typical object detection model.

(2) $$\mathrm{Precision}=\frac{True\;Positive}{True\;Positive+False\;Positive}$$

(3) $$\mathrm{Recall}=\frac{True\;Positive}{True\;Positive+False\;Negative}$$

Table 3 shows the precision, recall, AP, and F1-score values of the conventional Scaled YOLOv4 model and the proposed ensemble model as a function of the resolution of the MOT20Det data. In the case of the ensembled csp model, the difference in accuracy from the conventional model was not significant, but it showed an improvement in terms of FPS. The general accuracy of the ensemble model was strengthened as the resolution increased, and it had stronger detection performance for unfamiliar datasets despite having the same FPS. As the resolution increased, the precision and recall ratios were also close to 1, meaning that the training performance of the model was excellent.

**Figure 5:**
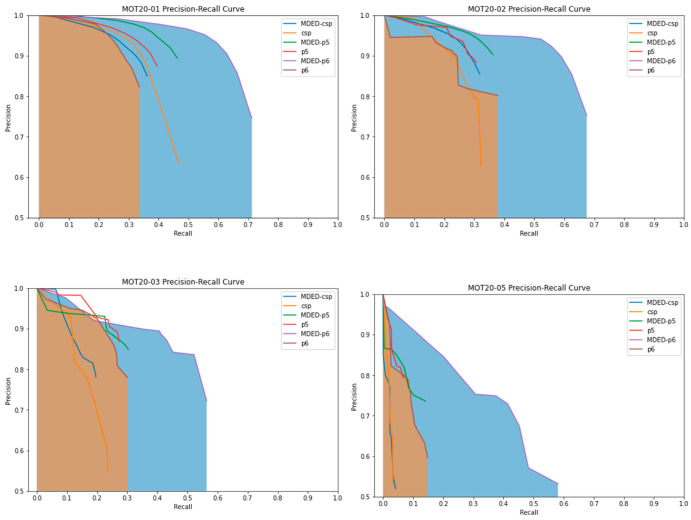
Precision–recall curves for the proposed model (MDED) and the Scaled YOLOv4 model.

Figure 5 shows the precision–recall curve of the MDED Framework deep learning model proposed in this paper and the comparison Scaled YOLOv4 model. Both precision and recall are evaluation indicators for which the closer to 1, the better the performance of the model, but the two indicators have an inverse relationship. Therefore, the more the graph is skewed to the upper right, the better the performance of the model can be evaluated. In addition, AP (average precision), which means the area under the precision–recall curve, is a common performance evaluation indicator for object detection. In Figure 5, we only show the AP for the P6 (1280) model, which had a high percentage of performance improvement. From Figure 5, we can see that overall, the MDED model is skewed to the upper right. This provides visual confirmation that the models generally perform well, even for data taken in different environments.

**Figure 6:**
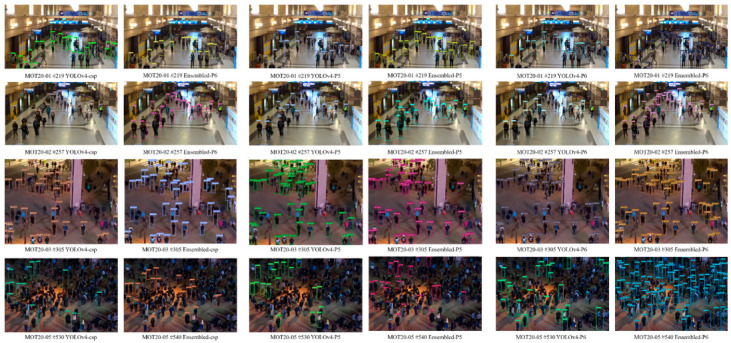
Comparison of MOT20Det detection results of the scaled YOLOv4 model and the proposed model as a resolution.

Figure 6 shows the detection results of the Scaled YOLOv4 model and the proposed framework on the MOT20Det dataset. The detection performance is better than that of the traditional model, despite the differences in indoor and outdoor settings, background contrast, and the density of pedestrian objects from the MOT17Det data used for training.

## 5. Conclusions and Future Works

The field of multi-object detection using deep learning models is still an active research area, and attempts to improve models operating in lightweight edge computing environments are ongoing. In this paper, we propose a pedestrian detection framework optimized for distributed processing in an edge computing environment, that can show improved performance with images other than the dataset it was trained on. The framework consists of Docker-based containers, and independent pipelines called preprocess microservices, inference microservices, and post-process microservices that are orchestrated through Kubernetes. This makes it easy to maintain the inference environment even as the edge computing environment changes; it also enables flexible scaling out and scaling down according to the quantity of resources available. By providing a web-based service that is familiar to users, we have created an environment where users can easily upload the videos they want to analyze and check the results.

Compared with the existing deep learning model (Scaled YOLOv4), the deep learning model improved by the proposed framework showed good performance in terms of accuracy and execution time. For an image with a resolution of 640, the performance was 2 FPS faster than the existing model; meanwhile, for an image with a resolution of 1280, the accuracy was up to 0.18 AP faster than the existing model. This shows that the proposed method can be used to obtain improved detection results in quasi-real time, even for unfamiliar data that have not been trained.

As part of our future research, we plan to assess the general performance of our model by utilizing the MOT17Det test and MOT20Det test datasets, which we were unable to use in this study due to authentication issues. This will allow us to compare our model’s accuracy with that of other models. Moreover, we intend to extend our microservice architecture to cover the entire training process, beyond the scope of the current paper that only covers the inference process. Specifically, we will incorporate a parameter server to enable deep learning model training in cloud-edge environments. Additionally, we will investigate and develop a framework to address the challenges of federated learning.

## Figures and Tables

**Table 1:** Specific information about the dataset used in the experiment.

Training Sequences						
Name	FPS	Resolution	Length	The Number of Pedestrian	Camera	Condition
MOT17-13-SDP	25	1920 × 1080	750 (00:30)	11,642	Moving	Day/outdoor
MOT17-11-SDP	30	1920 × 1080	900 (00:30)	9436	Moving	Indoor
MOT17-10-SDP	30	1920 × 1080	654 (00:22)	12,839	Moving	Night/outdoor
MOT17-09-SDP	30	1920 × 1080	525 (00:18)	5325	Static	Day/outdoor
MOT17-05-SDP	14	640 × 480	837 (01:00)	6917	Moving	Day/outdoor
MOT17-04-SDP	30	1920 × 1080	1050 (00:35)	47,557	Static	Night/outdoor
MOT17-02-SDP	30	1920 × 1080	600 (00:20)	18,581	Static	Day/outdoor
Total			5316 (03:35)	112,297		
**Testing Sequences**						
**Name**	**FPS**	**Resolution**	**Length**	**Number of** **Pedestrians**	**Camera**	**Condition**
MOT20-01	25	1920 × 1080	429 (00:17)	19,870	Static	Indoor
MOT20-02	25	1920 × 1080	2782 (01:51)	154,742	Static	Indoor
MOT20-03	25	1173 × 880	2405 (01:36)	313,658	Static	Night/outdoor
MOT20-05	25	1654 × 1080	3315 (02:13)	646,344	Static	Night/outdoor
Total			8931 (05:57)	1,134,641		

**Table 2:** Parameters and processing speeds of the deep learning models used in the experiment.

	Resolution	Params (B)	Layers	FPS
Scaled YOLOv4 (csp)	640	5.25	235	17
**HFN-csp (ours)**	640	**3.69**	**193**	**19**
**LFN-csp (ours)**	640	**3.13**	**191**	**19**
Scaled YOLOv4 (p5)	896	7.03	331	15
**HFN-p5 (ours)**	896	**5.13**	**281**	**16**
**LFN-p5 (ours)**	896	**5.66**	**309**	**16**
Scaled YOLOv4 (p6)	1280	12.7	417	14
**HFN-p6 (ours)**	1280	**9.5**	**367**	14
**LFN-p6 (ours)**	1280	**10.9**	**384**	14

**Table 3:** Precision, recall, AP, and F1-score results for the MOT20Det training data.

		MOT20-01	MOT20-02	MOT20-03	MOT20-05
Scaled YOLOv4 (csp)	Precision	0.63	0.63	0.55	0.29
	Recall	**0.47**	**0.32**	**0.24**	**0.07**
	*AP*_50_	**0.53**	0.37	0.25	0.05
	AP	**0.21**	0.15	0.08	0.02
	F1-score	**0.50**	0.37	**0.27**	**0.06**
**Ensembled** **(MDED-csp, ours)**	Precision	**0.85**	**0.86**	**0.78**	**0.52**
	Recall	0.36	0.32	0.20	0.04
	*AP*_50_	0.43	**0.39**	0.25	0.05
	AP	0.20	**0.18**	0.08	0.02
	F1-score	0.44	**0.40**	0.23	0.04
Scaled YOLOv4 (p5)	Precision	0.87	0.88	**0.87**	**0.79**
	Recall	0.40	0.31	0.27	0.07
	*AP*_50_	0.49	0.38	0.35	0.11
	AP	0.23	0.19	0.13	0.04
	F1-score	0.49	0.40	0.35	0.08
**Ensembled** **(MDED-p5, ours)**	Precision	**0.89**	**0.90**	0.85	0.74
	Recall	**0.46**	**0.36**	**0.30**	**0.14**
	*AP*_50_	**0.54**	**0.44**	**0.37**	**0.20**
	AP	**0.28**	**0.22**	**0.14**	**0.07**
	F1-score	**0.54**	**0.45**	**0.36**	**0.13**
Scaled YOLOv4 (p6)	Precision	**0.82**	**0.82**	**0.75**	**0.60**
	Recall	0.34	0.28	0.29	0.15
	*AP*_50_	0.45	0.36	0.34	0.18
	AP	0.20	0.16	0.12	0.06
	F1-score	0.41	0.36	0.36	0.16
**Ensembled** **(MDED-p6, ours)**	Precision	0.75	0.75	0.72	0.53
	Recall	**0.71**	**0.68**	**0.56**	**0.58**
	*AP*_50_	**0.76**	**0.74**	**0.60**	**0.48**
	AP	**0.37**	**0.35**	**0.19**	**0.16**
	F1-score	**0.70**	**0.67**	**0.56**	**0.50**

## Data Availability

The MOT17Det and MOT20Det datasets were utilized, which are available on the MOTChallenge site (https://motchallenge.net, accessed on 10 May 2023).
